# Hydrogel and scaffold strategies for talar osteochondral repair: biofabrication, ankle biomechanics, and clinical translation

**DOI:** 10.3389/fsurg.2026.1970956

**Published:** 2026-10-22

**Authors:** Qi Zhu, Zhengyu Liu, Beibei Chen, Jun Cui

**Affiliations:** 1 Department of Emergency, Central Hospital Affiliated to Shenyang Medical College, Shenyang, China; 2 Department of Foot and Ankle Surgery, Central Hospital Affiliated to Shenyang Medical College, Shenyang, China

**Keywords:** ankle biomechanics, biofabrication, cartilage repair, hydrogel, osteochondral lesion of the talus, regenerative medicine, scaffold, subchondral bone

## Abstract

Osteochondral lesions of the talus combine focal cartilage injury with variable subchondral bone damage in a small, highly loaded joint, making successful biomaterial translation dependent on more than regenerative capacity alone. This review examines hydrogel, scaffold, and biofabrication strategies for talar osteochondral repair through the combined lenses of ankle biomechanics, spatial construct design, and clinical feasibility. Defect containment, shoulder involvement, cystic depth, instability, and alignment alter the requirements for conformal filling, wet-field retention, cyclic compression and shear tolerance, and cartilage-bone anchorage. Recent biofabrication approaches, including gradient and multilayer scaffolds, 3D-printed architectures, decellularized extracellular-matrix hydrogels, directional microchannels, and adhesive or bioactive systems, provide increasing control over spatial organization and mechanical transitions, but most remain preclinical. Human studies support the feasibility of matrix- and scaffold-assisted talar repair, including autologous matrix-induced chondrogenesis, collagen- and hyaluronic acid-based platforms, cell-supported constructs, and related one-step procedures; however, heterogeneous lesion selection, concomitant bone grafting, instability correction, rehabilitation, imaging, and follow-up limit direct material-level comparisons. The central translational question is whether a material architecture addresses the dominant mechanical and surgical requirements of a defined talar lesion. Evaluation should therefore integrate wet-field handling, edge and interface stability, cyclic loading, bone-side integration, degradation under load, manufacturing reproducibility, and lesion-stratified clinical outcomes.

## 1 Introduction

Osteochondral lesions of the talus are difficult to treat because they combine focal cartilage injury, variable subchondral bone involvement, and a compact ankle contact environment. Treatment selection remains lesion-dependent because talar defects differ in size, containment, depth, cystic change, fragment viability, chronicity, and the presence of instability or malalignment (1, 2).

Bone marrow stimulation, fixation of viable fragments, osteochondral grafting, cell-based procedures, and matrix- or scaffold-assisted one-step repair are all used in clinical practice. Available comparative evidence has not identified a universally superior strategy across all talar lesion phenotypes (1–3). This uncertainty has encouraged interest in biomaterials that can improve local containment, support the cartilage-subchondral bone unit, and reduce the gap between early symptomatic improvement and durable structural repair.

Several recent reviews define important but complementary parts of this field. A 2024 systematic review and meta-analysis focused on the safety and clinical and radiological outcomes of scaffold-based treatment for osteochondral lesions of the talus (3), while a 2025 review surveyed gel-based scaffolds and templates across foot and ankle surgery (4). A 2023 systematic review specifically evaluated multilayered biomimetic talar scaffolds (5), and a 2026 review summarized advances in injectable hydrogels for talar osteochondral repair (6). These reviews primarily synthesize clinical outcomes or examine defined material classes. They do not, however, organize biomaterial design around the combined effects of quantitative tibiotalar mechanics, lesion containment and subchondral morphology, construct failure mode, and the preclinical tests needed to demonstrate suitability for a defined talar lesion.

The present review therefore uses a lesion-to-failure-mode-to-design-response framework rather than ranking scaffold products or cataloguing hydrogel chemistries. It links surface congruity and contact mechanics to controlled filling and seating; wet arthroscopic conditions and moving contact to retention and edge stability; repetitive compression and shear to fatigue resistance; and cystic or deep osteochondral involvement to bone-side support and graded cartilage-bone architecture. Evidence from non-talar preclinical models is considered only when it tests one of these design functions, not as proof of clinical efficacy in the ankle. Human studies are interpreted according to lesion size/depth, containment, subchondral reconstruction, instability or alignment, and comparator treatment. Figure 1 summarizes this ankle-specific framework.

**Figure 1:**
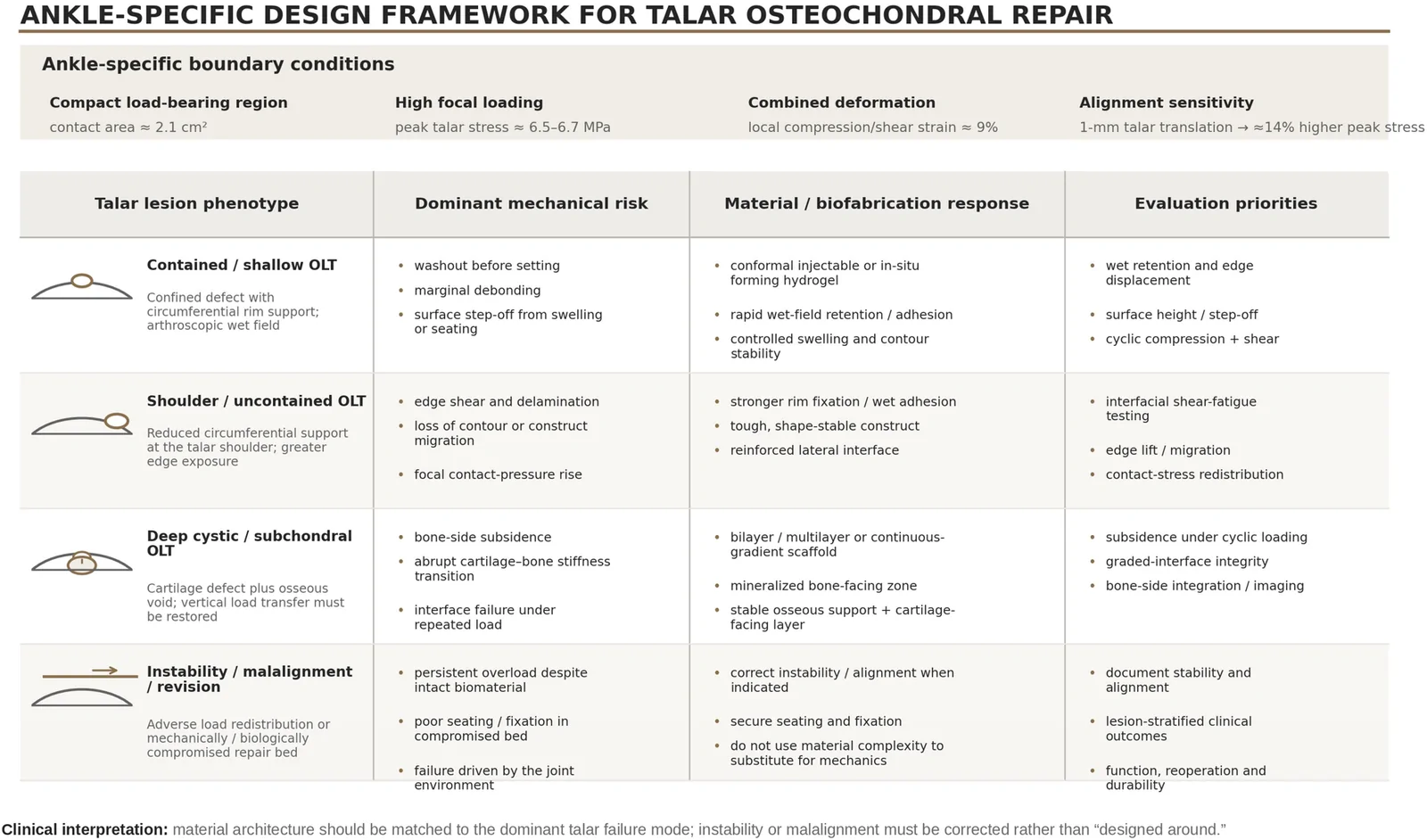
An ankle-specific lesion-to-design framework for hydrogel, scaffold, and biofabrication strategies in talar osteochondral repair. The upper band summarizes ankle-specific biomechanical boundary conditions as approximate joint-level values rather than material pass/fail thresholds. The matrix links contained/shallow, shoulder/uncontained, deep cystic/subchondral, and instability/malalignment/revision contexts to their dominant failure modes, material or biofabrication responses, and evaluation priorities. This mapping is talus-specific because it integrates surface congruity in a compact load-bearing region, wet-field retention, edge stability under combined compression and shear, bone-side support for cystic defects, and correction of adverse joint mechanics rather than treating osteochondral architecture alone as the design target.

A targeted search of PubMed-indexed literature was updated through 3 October 2026 using combinations of talus/talar/ankle, osteochondral lesion/defect, scaffold, matrix, hydrogel/gel, AMIC/MACI, biofabrication, 3D printing, gradient, multilayer, and related terms. Reference lists of recent talar scaffold, multilayer-scaffold, hydrogel, and ankle-repair reviews and eligible primary studies were also screened. For Table 1, original human studies reporting clinical outcomes after scaffold-, matrix-, or gel-assisted talar/ankle osteochondral repair were included; technical notes, single-case reports, reviews, and duplicate secondary analyses were excluded. Lesion size or depth, location/containment, subchondral reconstruction, instability/alignment, and comparator information were extracted when explicitly described; missing quantitative values were not imputed. Preclinical studies were selected when they addressed design functions relevant to talar translation, with biofabrication evidence sampled across continuous or zonal gradients, discrete biphasic/multilayer constructs, directional or spatial organization, and manufacturing/delivery/fixation strategies. Because this is a narrative translational review, the search was used to provide representative clinical and mechanistic coverage rather than a PRISMA-style systematic synthesis or meta-analysis.

**Table 1:** Primary human clinical studies directly reporting outcomes of scaffold-, matrix-, or gel-assisted talar/ankle osteochondral repair.

Study	Design	Material/strategy	Lesion phenotype/size	Subchondral/mechanical context/comparator	Main interpretation	Reference
Ronga et al.	Case series	Matrix-induced autologous chondrocyte implantation (MACI)	Deep ankle chondral defects; mean lesion area 3.4 cm^2^; five talar-dome lesions and one kissing lesion.	MACI for deep cartilage defects; no treatment comparator.	Early matrix-assisted chondrocyte experience in a small clinical series; no comparator.	(63)
Giannini et al.	Clinical cohort	Arthroscopic matrix-supported autologous chondrocyte implantation	Post-traumatic talar-dome osteochondral lesions (type II/IIA); 46-patient arthroscopic cohort.	Arthroscopic matrix-supported ACI; designed as an ankle-specific, minimally invasive approach; no comparator.	Reported clinical feasibility and improvement after arthroscopic matrix-supported chondrocyte implantation; noncomparative cohort.	(64)
Schneider and Karaikudi	Retrospective cohort	MACI grafting	Talar/ankle osteochondral defects treated with MACI; early clinical cohort without lesion-phenotype subgrouping.	Matrix grafting as a cartilage-restoration strategy; no separate instability/alignment or treatment comparator.	Reported substantial functional improvement after MACI; uncontrolled retrospective evidence.	(65)
Giannini et al.	Prospective clinical study	One-step bone marrow-derived cell transplantation with scaffold support	Talar osteochondral lesions; lesion area and depth were considered in outcome analysis in a 48-patient cohort.	One-step bone-marrow-derived cells with scaffold support and platelet gel; no parallel treatment control.	Reported feasibility of a one-step scaffold-supported cell strategy; treatment components were not separable.	(54)
Giza et al.	Prospective study	MACI	Full-thickness talar articular defects selected for MACI.	Matrix-induced ACI performed without malleolar osteotomy; no treatment comparator.	Prospective ankle-specific MACI study showing clinical improvement without malleolar osteotomy.	(66)
Dixon et al.	Clinical cohort	Matrix-associated autologous chondrocyte implantation	Ankle cartilage defects treated with matrix-associated ACI; cohort not organized by cystic/containment phenotype.	Cartilage-focused matrix-assisted reconstruction; no treatment comparator.	Reported functional improvement after matrix-associated chondrocyte implantation; noncomparative cohort.	(67)
Nehrer et al.	Long-term clinical/MRI cohort	Matrix-associated/autologous chondrocyte transplantation	Mixed ankle chondral/osteochondral defects with 2- to 11-year follow-up; heterogeneous lesion spectrum.	Matrix-associated or conventional chondrocyte transplantation; heterogeneous techniques preclude lesion-specific attribution.	Longer-term follow-up reported sustained clinical improvement with MRI assessment; techniques were heterogeneous.	(68)
Aurich et al.	Clinical/MRI cohort	Arthroscopic matrix-associated chondrocyte implantation	Arthroscopically treated ankle osteochondral lesions; lesion size was assessed as a clinical variable rather than used as a strict phenotype threshold.	Matrix-associated chondrocyte implantation with MRI follow-up; no treatment comparator.	Early arthroscopic matrix-associated treatment with parallel clinical and MRI assessment.	(69)
Pearce et al.	Case series	Synthetic biphasic osteochondral scaffold	Challenging talar osteochondral defects, including cystic or previously treated lesions for which a single-stage osteochondral option was sought.	Synthetic biphasic osteochondral graft providing cartilage- and bone-side reconstruction; no control group.	Early clinical experience with a synthetic osteochondral plug; small uncontrolled series.	(70)
Magnan et al.	Mid-term cohort	Three-dimensional matrix-induced autologous chondrocyte implantation	Outerbridge grade III–IV talar OLTs; inclusion 1.5–4.0 cm^2^, mean 2.36 cm^2^; predominantly medial lesions.	3D matrix-induced ACI; chronic lateral instability was present in six patients and represents an important mechanical modifier.	Mid-term clinical series supporting 3D matrix-assisted chondrocyte implantation; no randomized comparator.	(71)
Anders et al.	5-year cohort	Matrix-associated autologous chondrocyte implantation	Full-thickness chondral/osteochondral talar lesions; mean 1.94 cm^2^ (range 1–6 cm^2^).	Matrix-associated ACI; 13 osteochondral defects received simultaneous autologous bone grafting; no comparator.	Five-year follow-up reported maintained clinical improvement after matrix-associated chondrocyte implantation in deep lesions.	(72)
Giannini et al.	4-year follow-up cohort	One-step bone marrow-derived cell/scaffold repair	Mid-term follow-up of the one-step talar OLT cohort, with lesion morphology/size retained as clinically relevant variables.	Bone-marrow-derived cell/scaffold repair with T2 mapping; no parallel comparator.	Clinical improvements were maintained at mid-term; MRI T2 mapping provided additional structural assessment.	(73)
Lee et al.	Clinical/second-look cohort	Fibrin matrix-mixed gel-type autologous chondrocyte implantation	Talar osteochondral lesions treated with gel-type ACI and evaluated clinically and at second-look arthroscopy.	Fibrin matrix-mixed gel-type ACI; no separate treatment comparator.	Combined clinical and second-look arthroscopic assessment of gel-type chondrocyte implantation.	(74)
Valderrabano et al.	Clinical cohort	Autologous spongiosa graft plus AMIC	Osteochondral talar defects requiring combined cartilage and osseous reconstruction; 26-patient cohort.	Autologous spongiosa graft + AMIC; concomitant ligament repair in 17/26 and corrective calcaneal osteotomy in 16/26, highlighting instability/alignment correction.	Combined cartilage matrix and bone reconstruction was associated with clinical improvement; matrix and graft effects cannot be isolated.	(75)
Walther et al.	Clinical series	Collagen-matrix cartilage reconstruction	Focal talar cartilage defects selected for miniarthrotomy collagen-matrix reconstruction.	Cartilage-focused collagen-matrix procedure; no dedicated cystic-depth or instability subgroup analysis.	Described a miniarthrotomy collagen-matrix approach with clinical outcome reporting.	(76)
Giannini et al.	Clinical cohort	Arthroscopic scaffold-based autologous chondrocyte implantation	Talar/ankle cartilage and osteochondral lesions in an extended arthroscopic chondrocyte-implantation cohort.	Arthroscopic scaffold-based ACI; no parallel treatment comparator.	Expanded clinical experience with arthroscopic scaffold-based chondrocyte implantation.	(77)
Volpi et al.	Clinical cohort	Autologous collagen-induced chondrogenesis (ACIC)	Small preliminary series of talar chondral lesions.	Microfracture plus injectable atelocollagen (ACIC); cartilage-focused augmentation without a separate control arm.	Introduced injectable atelocollagen augmentation combined with microfracture in a clinical talar series.	(78)
Buda et al.	Comparative cohort	One-step bone marrow-derived cells on scaffold	Talar osteochondral lesions treated with regenerative procedures; comparative clinical cohort.	One-step bone-marrow-derived cells on scaffold vs. autologous chondrocyte implantation; treatment packages, not isolated materials, were compared.	Both regenerative approaches improved outcomes; nonrandomized comparison limits treatment ranking.	(55)
Christensen et al.	Clinical/radiological cohort	Biomimetic multilayer collagen scaffold	Mixed knee/talus osteochondral-defect cohort with a small talar subgroup.	Biomimetic multilayer collagen scaffold; talar-specific effects cannot be separated reliably from the mixed cohort.	Reported limited osteochondral repair quality; talar numbers were small and results should not be generalized.	(79)
Usuelli et al.	1-year clinical/MRI cohort	Autologous collagen-induced chondrogenesis (ACIC)	Talar osteochondral lesions; mean lesion area 2.1 ± 0.9 cm^2^ in the reported cohort.	All-arthroscopic autologous collagen-induced chondrogenesis with MRI assessment; no parallel comparator.	One-year clinical and MRI evaluation supported feasibility of injectable collagen-assisted repair.	(80)
Kubosch et al.	Clinical/MRI cohort	AMIC	Medial talar osteochondral lesions treated as combined cartilage-subchondral defects.	Autologous cancellous bone graft + AMIC with T2 mapping; no treatment comparator.	Clinical improvement was reported with imaging assessment of repair tissue; no randomized control.	(81)
Kaipel et al.	Clinical/MRI cohort	Cell-free multilayered nano-composite scaffold	Medial talar osteochondral defects ≥1.5 cm^2^ in a four-patient clinical/MRI cohort.	Cell-free multilayered nanocomposite osteochondral scaffold; no comparator.	Clinical improvement contrasted with limited tissue quality on advanced MRI, illustrating clinical-structural discordance.	(82)
Sadlik et al.	Mid-term cohort	Modified sandwich reconstruction with scaffold and bone support	Large osteochondral lesions of the talar dome requiring structural reconstruction.	Modified sandwich technique combining scaffold/cartilage repair with bone support; no isolated scaffold comparator.	Reported outcomes after reconstruction of large lesions; the combined procedure prevents attribution to one component.	(83)
Gottschalk et al.	Prospective 5-year cohort	AMIC	Talar osteochondral lesions; mean defect area 1.4 ± 0.9 cm^2^ (range 0.2–4.0 cm^2^).	AMIC; prospective single-cohort evidence without a treatment comparator.	Pain, function, and activity improved at mid-term; single-cohort evidence.	(84)
Albano et al.	Clinical/MRI cohort	AMIC with biomimetic scaffold	Talar osteochondral lesions in a small clinical/MRI cohort.	AMIC combined with a biphasic biomimetic osteochondral scaffold; no treatment comparator.	Clinical improvement was accompanied by imaging assessment; small nonrandomized cohort.	(85)
Kanatli et al.	Clinical/radiological cohort	Microfracture plus cell-free polymer scaffold	Talar OLTs ≥1.5 cm^2^; mean area 2.5 ± 0.8 cm^2^ and mean volume 2.4 ± 1.9 cm^3^.	Microfracture + cell-free polymer scaffold; bone graft added for cyst depth >10 mm; lower-extremity deformity excluded.	Reported satisfactory clinical and radiological results; no randomized comparator.	(86)
Tahta et al.	Retrospective comparative study	Hyaluronic acid scaffold plus concentrated bone marrow aspirate	Focal talar OLTs in a 98-patient comparative cohort; lesion dimensions were comparable between groups at baseline.	Hyaluronic-acid scaffold + concentrated BMAC vs. nanofracture; combined scaffold/cell-concentrate effect cannot be isolated.	Scaffold/BMAC group showed better clinical and MOCART outcomes; retrospective design and combined biologic treatment limit causal attribution.	(87)
D'Ambrosi et al.	Juvenile cohort	Microfracture plus autologous bone graft and AMIC	Juvenile talar OLTs; mean intraoperative lesion volume 1.1 ± 0.5 cm^3^; baseline lesion area approximately 119 mm^2^ on CT and 132 mm^2^ on MRI.	Microfracture + autologous bone graft + AMIC; combined cartilage-subchondral reconstruction; no control arm.	Feasible combined osteochondral reconstruction in younger patients; no control arm.	(88)
Usuelli et al.	Clinical/radiological cohort	All-arthroscopic AMIC plus autologous bone graft	Type III–IV talar osteochondral defects; mean area 111.1 ± 43.2 mm^2^ on CT and 154.1 ± 93.6 mm^2^ on MRI.	Autologous bone graft in all cases + all-arthroscopic AMIC; no associated procedure at index surgery and no comparator.	Reported outcomes of an all-arthroscopic matrix-plus-bone strategy; graft contribution cannot be separated from matrix effect.	(26)
Baumfeld et al.	Short-term case series	All-arthroscopic AMIC	Symptomatic talar osteochondral defects selected for an all-arthroscopic AMIC procedure.	All-arthroscopic matrix-induced chondrogenesis; short-term single-cohort evidence without a comparator.	Short-term all-arthroscopic AMIC experience; small uncontrolled cohort.	(89)
Kreulen et al.	7-year follow-up cohort	MACI	Seven-year follow-up of the earlier talar MACI cohort rather than a newly selected lesion-phenotype cohort.	Long-term durability assessment of MACI; lesion characteristics derive from the original cohort and there is no comparator.	Extended the earlier MACI cohort to 7 years, providing durability data from an overlapping patient series.	(90)
Yontar et al.	Clinical cohort	Microfracture plus cell-free hyaluronic acid scaffold	Small-area but deep talar OLTs: <1.5 cm^2^ and >7 mm deep (mean depth 10.4 ± 1.9 mm).	Microfracture + cell-free hyaluronic-acid scaffold; designed for deep osteochondral morphology; no parallel comparator.	Reported favorable clinical outcomes with one-step scaffold augmentation; uncontrolled design.	(91)
Eren et al.	Comparative cohort	Cell-free scaffold implantation	Talar OLTs in a comparative cohort; scaffold implantation was evaluated particularly as an alternative for larger lesions.	Cell-free scaffold implantation vs. microfracture; nonrandomized allocation limits causal comparison.	Both approaches improved outcomes; scaffold implantation showed advantages in selected measures, but allocation was not randomized.	(92)
Weigelt et al.	2- to 8-year cohort	AMIC	Medial talar-dome OLTs; mean area 0.9 ± 0.5 cm^2^ (range 0.4–2.3 cm^2^).	AMIC with autologous bone graft in 28/33; patients requiring additional procedures were excluded, reducing mechanical confounding.	Sustained clinical benefit was reported, while structural repair quality varied.	(29)
Galla et al.	Clinical cohort	Autologous spongiosa graft plus matrix-induced chondrogenesis	Talar osteochondral lesions with subchondral involvement requiring osseous reconstruction.	Autologous spongiosa graft + matrix-induced chondrogenesis; open reconstruction performed without medial malleolar osteotomy.	Reported clinical outcomes of combined bone/matrix reconstruction using an alternative surgical approach.	(30)
Becher et al.	Comparative study	Arthroscopic AMIC	Matched talar articular-cartilage defects treated by two marrow-stimulation strategies.	Arthroscopic AMIC vs. arthroscopic microfracture; direct treatment comparison, with lesion characteristics balanced rather than separately stratified.	No uniform superiority across all outcomes; supports cautious interpretation of matrix augmentation.	(93)
Richter et al.	Prospective 5-year cohort	Matrix-associated stem cell transplantation (MAST)	Ankle chondral lesions treated within a 100-patient complex reconstructive cohort.	Matrix-associated stem-cell transplantation with concomitant procedures; mechanical corrections and adjunct procedures limit attribution to MAST alone.	Large noncontrolled cohort with durable improvement; concomitant procedures limit attribution to MAST alone.	(94)
Murphy et al.	Clinical cohort	Matrix-associated stem cell transplantation (MAST)	Larger or previously treatment-resistant talar osteochondral lesions; 38 lesions in 32 patients.	Matrix-associated stem-cell transplantation; no parallel comparator and no isolated subchondral-material effect.	An ankle-specific MAST cohort reported favorable clinical outcomes; comparative evidence remains limited.	(95)
Lee et al.	Multicenter randomized trial	Microfracture plus atelocollagen augmentation	Talar OLTs suitable for arthroscopic marrow stimulation in a multicenter randomized trial.	Microfracture in both groups, with atelocollagen augmentation in the intervention arm; direct adjunct-vs.-control comparison.	Randomized trial found higher 2-year MOCART scores with atelocollagen augmentation, while between-group clinical score differences were not statistically significant.	(58)
Camurcu et al.	Comparative study	Microfracture plus chitosan/blood implant	Talar OLTs treated with arthroscopic microfracture in a comparative clinical/MRI study.	Microfracture + chitosan/blood implant vs. microfracture alone; direct adjunct comparison.	Adjunctive chitosan/blood implantation did not consistently improve clinical or MRI outcomes.	(59)
Akmese et al.	Comparative cohort	Chitosan-based liquid scaffold	Talar osteochondral lesions treated with cell-free scaffold strategies in an 81-patient comparative cohort.	Chitosan-based liquid scaffold vs. hyaluronic-acid-based soft scaffold; direct scaffold-to-scaffold comparison.	Both scaffold types improved clinical outcomes without a clear between-scaffold advantage.	(96)
Gotze et al.	5-year cohort	AMIC with cancellous bone graft	Focal talar shoulder defects, an uncontained/high-edge-shear lesion phenotype.	AMIC with cancellous bone graft; shoulder location provides a mechanically demanding context; no control group.	Five-year cohort reported clinical improvement after AMIC with cancellous bone graft in talar shoulder defects.	(97)
Richter et al.	Matched-patient analysis	MAST vs. AMIC plus peripheral blood concentrate	Ankle chondral lesions treated in a matched-patient complex-surgery setting.	MAST vs. AMIC + peripheral blood concentrate; concomitant procedures remain part of both treatment packages.	Direct matched comparison of two matrix-associated strategies within a complex surgical setting.	(98)
Lenz et al.	Long-term cohort	MACI grafting	Talar osteochondral lesions in a long-term MACI cohort.	MACI durability assessment; no parallel control and no dedicated lesion-phenotype subgroup comparison.	Long-term follow-up reported maintained improvement after MACI in a small selected cohort.	(99)
Ayyaswamy et al.	Retrospective cohort	AMIC	Predominantly larger talar OLTs; mean lesion diameter 1.74 cm, 88% > 1.4 cm, maximum 2.5 cm.	AMIC in a retrospective cohort; no parallel comparator.	Clinical improvement reported; heterogeneity and absence of a control group limit comparative inference.	(27)
Migliorini et al.	Prospective cohort	AMIC	Focal unipolar talar shoulder defects; mean area 2.7 cm^2^, with 2–3 cm^2^ used for cohort selection.	AMIC vs. microfracture; cancellous graft used when subchondral bone was nonviable; kissing/multifocal lesions and skeletal malformation excluded.	AMIC was associated with better clinical scores and lower reoperation/failure rates than microfracture; MOCART did not differ significantly.	(28)
Dogar et al.	Multicenter prospective randomized trial	Arthroscopic treatment strategies including scaffold/matrix adjuncts	Talar osteochondral lesions enrolled in a multicenter prospective randomized arthroscopy trial.	Randomized comparison of arthroscopic treatment strategies including scaffold/matrix adjuncts; interpretation depends on the specific randomized arm.	Randomized comparison across arthroscopic strategies; interpretation depends on the specific treatment components and lesion selection.	(60)
Drobnic et al.	Early case series	Biphasic aragonite osteochondral implant	Chronic deep Hepple grade 4–5 OLTs, 1.8–2.2 cm^2^; three medial-central lesions and one combined medial/lateral lesion.	Press-fit biphasic aragonite osteochondral implant; surgical exposure adapted to lesion location; four-patient feasibility series.	Four-patient pilot series supports technical feasibility in complex lesions; it does not establish efficacy.	(61)
Ackermann et al.	Matched cohort	AMIC with or without lateral ligament stabilization	Talar OLTs in patients with chronic ankle instability.	AMIC with vs. without concomitant lateral ligament stabilization; directly tests correction of the mechanical environment.	Residual instability was associated with worse clinical outcomes, emphasizing correction of the mechanical environment.	(100)
Bajuri et al.	Small case series	Microfracture plus cell-free hyaluronic acid scaffold	Large grade III–IV talar osteochondral lesions in a very small clinical series.	Microfracture + cell-free hyaluronic-acid scaffold; no parallel comparator.	Functional improvement reported after Hyalofast-assisted repair; very small uncontrolled series.	(101)
Cengiz and Moradi	Comparative study	Microfracture plus acellular scaffold	Larger talar osteochondral lesions, approximately 1.5–3.0 cm^2^ in the reported cohort.	Arthroscopic microfracture ± acellular scaffold; direct scaffold-augmentation comparison.	Both strategies improved outcomes; scaffold augmentation was evaluated as an adjunct rather than an isolated treatment.	(102)
Körner et al.	Retrospective case series	Matrix-associated autologous chondrocyte implantation plus bone graft	Adolescent talar OLTs; median lesion area 1.3 cm^2^ (range 0.9–3.2 cm^2^) and median depth 5 mm (range 5–9 mm).	Matrix-associated ACI + autologous bone graft; combined cartilage-subchondral reconstruction; no comparator.	Provides adolescent long-term patient-reported outcomes for combined cartilage and subchondral reconstruction.	(103)
Gottschalk et al.	Registry cohort	M-BMS plus type I/III collagen scaffold	Medial talar OLTs; mean lesion area about 1.7 ± 1.3 cm^2^ and volume about 1.0 ± 1.38 cm^3^ in registry reporting.	M-BMS + type I/III collagen scaffold; medial malleolar osteotomy evaluated as a surgical modifier.	Registry data assessed scaffold-assisted repair with attention to medial malleolar osteotomy as a surgical modifier.	(104)
Migliorini et al.	Revision cohort	Repeat AMIC	Recurrent symptomatic OLT after failed prior AMIC; revision-lesion phenotype.	Repeat AMIC in a revision setting; no untreated or alternative-treatment control.	Shows that matrix-assisted repair can be used in revision settings; selected cohort and no control.	(31)
Yontar et al.	Clinical cohort	Autologous matrix-related chondrogenesis	Large talar OLTs >150 mm^2^; cohort included primary, tumor-related, and revision lesions.	Autologous matrix-related chondrogenesis; subgroup comparisons reflect lesion etiology/revision status rather than a separate treatment control.	Reported functional improvement in large lesions; uncontrolled cohort.	(105)
Kekec and Yildirim	Comparative study	Arthroscopic AMIC with or without scaffold	Deep Bristol stage 4–5 talar OLTs, 1.5–3.0 cm^2^; mean area 2.12 ± 0.32 cm^2^ and mean depth about 11 ± 4 mm; cystic morphology was characteristic.	Curettage/microfracture with grafting; PGA-HA scaffold added in one group; patients with concomitant ligament injury were excluded.	Directly evaluates the contribution of scaffold use in deep lesions; mid-term nonrandomized evidence.	(32)
Vannini et al.	Long-term cohort	Hyaluronic scaffold plus bone marrow concentrate	Talar OLTs; mean lesion area 2.2 ± 1.4 cm^2^; predominantly post-traumatic lesions.	Hyaluronic scaffold + BMAC; prior ankle fracture and osteoarthritic change were present in subsets; no parallel control.	Durable improvement was reported in selected patients; long-term cohort without randomized comparator.	(56)
Gorgun et al.	Comparative cohort	Collagen scaffold plus autologous cancellous bone graft	Talar OLTs with concomitant subchondral bone defects requiring cancellous bone reconstruction.	Debridement + microfracture + autologous cancellous bone graft in both groups; collagen scaffold added in the intervention group.	Clinical improvement did not differ with scaffold addition; MOCART was slightly lower in the scaffold group, emphasizing the role of bone reconstruction.	(62)
Tan et al.	Comparative clinical study	Atelocollagen-induced chondrogenesis	Talar osteochondral lesions treated with a marrow-stimulation-based strategy in a short-term comparative cohort.	Microfracture + atelocollagen-induced chondrogenesis vs. microfracture alone; direct adjunct comparison.	Propensity-matched comparison found similar 1-year clinical score improvement; satisfaction and several quality-of-life domains favored atelocollagen augmentation.	(57)
Deiss et al.	10-year cohort	AMIC	Talar OLTs; mean defect area 1.4 ± 0.9 cm^2^ (range 0.2–4.0 cm^2^) in the 10-year cohort.	Long-term AMIC follow-up; no parallel comparator.	Patient-reported improvement remained durable at 10 years; cohort design and evolving technique limit generalization.	(33)
Peras et al.	Retrospective multicenter cohort	AMIC	SFA stage 3 talar-dome lesions; mean defect area 1.83 cm^2^ (range 0.6–6.0 cm^2^).	AMIC in a retrospective multicenter cohort; multicenter technique variation is an important contextual modifier.	Multicenter retrospective cohort reported improved clinical scores; selection and technique heterogeneity limit comparative inference.	(106)
Proietti et al.	Activity-outcome cohort	Arthroscopic AMIC	Sports-active patients with talar OLTs >1.5 cm^2^.	Arthroscopic AMIC; lesion bed debrided and osseous deficiency reconstructed as required before membrane fixation; return-to-sport focus, no comparator.	Return to sport was reported in selected patients; structural durability was not the primary endpoint.	(107)
Richter et al.	Prospective 7-year cohort	AMIC plus peripheral blood concentrate	Mixed ankle chondral lesions, predominantly talar; average lesion area about 1.8 cm^2^; medial/lateral shoulder locations both represented.	AMIC + peripheral blood concentrate within complex reconstructive surgery; concomitant procedures limit isolation of the matrix effect.	Longer-term clinical experience with biologic augmentation; concomitant procedures limit isolation of the matrix effect.	(108)
Efrima et al.	5-year cohort	Arthroscopic AMIC	Talar OLTs analyzed by lesion-size strata of 1–1.5 and >1.5 cm^2^.	Arthroscopic AMIC; lesion size was evaluated as a potential outcome modifier rather than treated as a single uniform phenotype.	Sustained clinical improvement reported at 5 years; selected cohort.	(109)
Khattab et al.	Pilot study	AMIC	Symptomatic talar OLTs >150 mm^2^, Berndt grade ≥2; predominantly posteromedial lesions in the pilot cohort.	AMIC after failed conservative/marrow-stimulation options; bone graft and medial malleolar osteotomy used according to osseous depth/location; no comparator.	Pilot data support feasibility; sample size and follow-up limit inference.	(110)
Baumfeld et al.	Mid-term case series	All-arthroscopic AMIC	Talar OLTs; mean lesion area 112 mm^2^; Raikin zone 4 was the most common location (about 55%).	All-arthroscopic microfracture + collagen matrix; autologous cancellous bone graft used when subchondral reconstruction was required.	Provides recent all-arthroscopic outcome data; no comparative arm.	(111)
Gatti et al.	Retrospective case series	Arthroscopic autologous collagen-induced chondrogenesis	Hepple grade II-IV talar OLTs; mean defect area 76.6 ± 33.6 mm^2^ (range 35–135 mm^2^).	Arthroscopic ACIC + microfracture; concomitant intra-articular pathology addressed at the same operation; no parallel comparator.	Recent injectable collagen-based clinical experience; retrospective single-cohort evidence.	(112)
Baumfeld et al.	Retrospective case series	Autologous platelet-rich fibrin biological scaffold (A-PRF + i-PRF)	Symptomatic talar OLTs in a 36-patient cohort; lesion-size/depth threshold was not specified.	Arthroscopic lesion preparation with an intraoperatively prepared PRF scaffold; no parallel comparator.	Pain and functional scores improved and >80% returned to sport; the uncontrolled design does not establish the incremental benefit of PRF.	(135)

Lesion phenotype/size, subchondral treatment, instability/alignment or other mechanical context, and comparator information are presented where reported to support lesion-specific interpretation of the clinical evidence. ACI, autologous chondrocyte implantation; AMIC, autologous matrix-induced chondrogenesis; BMAC, bone marrow aspirate concentrate; MACI, matrix-induced autologous chondrocyte implantation; MRI, magnetic resonance imaging; OLT, osteochondral lesion of the talus. Lesion-level fields summarize information explicitly described in the primary report or its clearly linked cohort. When an exact numerical value was not provided, the reported phenotype and use of the variable for selection/stratification are described instead; no missing quantitative values were imputed. Because many procedures combined a matrix or scaffold with marrow stimulation, bone grafting, cells, or concomitant stabilization, observed outcome changes should not be attributed to the material component alone.

## 2 Ankle-specific biomechanical parameters and material requirements

Weightbearing and *in vivo* studies provide several measurable parameters that are more informative than a generic description of the ankle as a high-load joint. In a bilateral weightbearing CT-based computational study of healthy ankles, mean talar contact stress was approximately 1.9–2.0 MPa, peak talar contact stress approximately 6.5–6.7 MPa, and mean tibiotalar contact area approximately 2.1 cm^2^ (8). In an MRI-based digital volume correlation study under compressive loading, talar and distal tibial translations were approximately 1 mm and local compressive and shear contact strains approached 9%; 87.5% of peak tensile, compressive, and shear strains occurred in the medial or lateral talar zones (7). These measurements should not be interpreted as universal material targets because loading protocols and modeling assumptions differ between studies. Rather, they define ankle-specific boundary conditions against which a construct can be assessed: preservation of contact area and surface congruity, avoidance of focal stress concentration, and maintenance of stability during combined compression and shear.

The sensitivity of tibiotalar mechanics to relatively small changes in alignment further narrows the acceptable mechanical envelope. A weightbearing CT-based finite-element analysis predicted that 1 mm of lateral talar translation increased peak talar contact stress by 14% and reduced contact area by 3%, while external rotation greater than 12 degrees produced the largest effect on predicted peak contact stress (9). In a cadaveric ankle model, fibular malrotation increased peak contact pressure in neutral position from 5.56 to 7.21 MPa under the tested loading condition, although the direction and magnitude of the change varied with ankle position (10). These findings indicate that implant performance cannot be separated from joint congruity: a biomaterial cannot compensate for persistent instability or malalignment, and experimental evaluation should distinguish intrinsic construct failure from mechanically adverse boundary conditions created by the ankle itself.

These joint-level parameters translate into interface-level material requirements. At the articular surface, a construct should preserve contour and avoid swelling- or seating-related step-off that could concentrate load. At the cartilage rim, wet adhesion and interfacial shear resistance are required to limit edge separation during motion. At the bone-facing side, deep or cystic defects require resistance to subsidence and adequate vertical load transfer. For hydrogels, a single compressive modulus is therefore insufficient; swelling-related height change, wet-field retention, edge displacement, cyclic recovery, and interfacial failure should also be reported. For structured scaffolds, seating accuracy, fatigue deformation, bone-side support, and integrity of graded or layered interfaces are similarly relevant.

Accordingly, an ankle-specific preclinical test matrix should report, where feasible, construct height or surface step-off before and after setting or swelling; changes in contact area and stress distribution relative to an intact or reconstructed baseline; retention and edge displacement in a hydrated field; cyclic compressive and shear deformation or failure; and bone-side subsidence or interface failure for osteochondral constructs. These are evaluation variables rather than validated pass/fail thresholds. Current ankle literature does not define a single minimum modulus, adhesive strength, or fatigue limit that guarantees clinical success. Evidence from non-talar models is therefore most useful when it tests candidate solutions to these ankle-defined failure modes, rather than when it is treated as direct evidence of talar efficacy.

Clinical experience reinforces this lesion-specific interpretation. Long-term and predictor studies of marrow stimulation report symptomatic improvement, but durability varies (11, 12), and outcomes are influenced by lesion size and other lesion- and patient-level factors (13, 14). These observations do not provide material thresholds; instead, they identify the clinical context that determines whether a scaffold or hydrogel needs mainly contour retention, edge stability, or additional subchondral support.

More recent cohorts add detail to this clinical picture. Small or shallow cysts do not necessarily compromise microfracture outcomes (15). Ten-year survival data indicate that a substantial proportion of patients remain free from revision at long-term follow-up (16). Smoking, postoperative bone marrow edema, second-look cartilage quality, and drilling-related vascular disturbance further emphasize the importance of the osteochondral unit rather than cartilage alone (17–20). Return-to-sport studies also show why activity recovery should be considered separately from structural repair (21).

## 3 Lesion phenotype as a design constraint

Clinical presentation is often nonspecific, with persistent pain, swelling, catching, recurrent effusion, or activity limitation after trauma or repetitive overload (1, 2). MRI and CT are therefore important for defining lesion location, containment, cartilage integrity, cystic change, and subchondral depth (22–25). These features, together with instability and alignment, determine the mechanical problem that a repair material must address.

The dominant mechanical requirement differs among contained, shoulder or uncontained, and deep cystic lesions. Small contained defects place greater emphasis on conformal filling, surface congruity, and retention at hydrated margins. Shoulder or uncontained lesions have less circumferential support and therefore place greater emphasis on edge stability and resistance to shear-related delamination. Deep cystic lesions require restoration of bone-side support and vertical load transfer in addition to cartilage-surface reconstruction (1, 2). Arthroscopic AMIC combined with bone grafting illustrates the need to reconstruct the osseous component when subchondral involvement is substantial (26). Clinical series of AMIC likewise show that outcomes vary with lesion location, depth, prior treatment, surgical approach, and duration of follow-up (27–33).

These differences limit broad claims about material performance. An injectable adhesive hydrogel may be suitable for a small contained defect but remains incomplete for a deep cystic lesion unless bone-side support is addressed. A mineralized or bilayer scaffold may be more relevant when the subchondral bone is involved, but it still has to be trimmed, seated, and secured without creating a proud surface. Instability or malalignment can alter contact mechanics independently of the implanted material (7–10).

Material selection should therefore follow the dominant failure risk of the lesion. In a compact contained defect, delivery and retention may be the main concerns. In a deep lesion, restoration of bone-side support and a graded osteochondral transition becomes more important. Revision lesions add the challenge of a mechanically and biologically compromised bed.

Preclinical studies would be more informative if they reported how a construct fails as well as whether it improves histology or compressive modulus. Plausible early failure modes for testing include washout during implantation, loss of contour before setting, debonding from hydrated cartilage, surface incongruity after swelling or degradation, delamination under cyclic shear, fatigue cracking within the soft phase, and collapse on the bone-facing side.

A practical preclinical sequence is to assess wet-field delivery and early retention before more complex biological functions, because adhesion, washout resistance, and cartilage-edge bonding are immediate translational barriers (34–37). Cyclic compression and shear can then be tested in a hydrated environment to capture repetitive-loading failure (38, 39). Bone-side support and mineral gradients should likewise be examined under repeated loading rather than by bulk stiffness alone (40, 41). Marginal sealing, spatially patterned biological cues, and mechanical gradients further illustrate why interfacial behavior should be reported alongside swelling, viability, and other biological readouts (42–44).

Imaging and mechanical studies also indicate which lesion features and repair variables should be measured. Localization grids and MRI or cone-beam CT staging define defect position, containment, and depth (22–25). Surface congruity deserves separate assessment. In cadaveric talar osteochondral grafting, flush graft placement restored near-normal contact pressure, whereas 0.5- or 1.0-mm elevation increased local graft-site pressure and recession shifted load away from the graft site; the magnitude of these effects also differed between medial and lateral lesions (45). By contrast, a finite-element analysis in which the graft surface remained flush found that using a thicker knee-derived cartilage layer changed mean tibiotalar contact pressure only minimally compared with a thickness-matched graft (46). Together, these findings suggest that surface step-off and seating may be more immediate mechanical hazards than cartilage-thickness mismatch alone, supporting explicit reporting of construct height, swelling-related contour change, and edge seating. Long-term marrow-stimulation and cyst-comparison cohorts further indicate that follow-up duration, cyst status, and outcome timing should not be collapsed into a single repair endpoint (47–50).

## 4 Material, biofabrication, and clinical strategies

### 4.1 Conformal filling, wet-field retention, and fatigue resistance

For injectable or *in situ* forming materials, delivery is an immediate practical constraint. The material must enter a small, irregular, wet talar defect without uncontrolled spread, incomplete filling, or early loss. In a large-animal cartilage model, a photocrosslinkable hydrogel cured rapidly under water-filled arthroscopic conditions, resisted washout, and supported cartilage repair (34). Although the model was not ankle-specific, it addresses the need to maintain control of the material during arthroscopic implantation before biological integration occurs.

Retention at the defect margin is the next challenge. Hydrated cartilage is difficult to bond, and a construct with acceptable bulk stiffness can still fail if it separates from the surrounding rim. GelMA-glycol chitosan constructs, chemically anchored hyaluronic acid systems, and enzyme-assisted GelMA bonding have improved cartilage-hydrogel coupling in preclinical or *ex vivo* models (35–37). For talar applications, the relevance of these studies lies mainly in the host-construct interface. Edge bonding should therefore be tested under load rather than inferred from bulk material properties.

After initial fixation, repetitive loading becomes central. Walking, progressive rehabilitation, and return to sport expose the ankle to repeated compression and shear. Hydrogels engineered for rapid recovery and fatigue resistance show that endurance can be incorporated into soft repair platforms (38, 39). A single compressive modulus is therefore an incomplete measure for talar use. Repeated deformation, shear challenge, wet handling, and failure at the cartilage edge or osteochondral interface provide more clinically relevant information.

Degradation also needs to be considered in relation to postoperative support. Rapid resorption may be useful when tissue replacement is fast, but premature loss of material can leave the repair site unsupported before incorporation is mature. Prolonged persistence can create a different problem if it impedes remodeling or maintains a marked stiffness mismatch. Degradation data are therefore most useful when reported together with retention, load support, and interfacial failure.

### 4.2 Osteochondral anchorage and graded interfaces

Because talar osteochondral lesions involve both cartilage and subchondral bone, repair must also restore a functional transition between these tissues. An abrupt change in structure or stiffness can concentrate shear at the interface and predispose the repair to delamination. Continuous-gradient mineralized hydrogels and nanohydroxyapatite-gradient scaffolds are designed to distribute this transition more gradually (40, 41).

Bone-side anchorage and lateral sealing need to be considered together. Marginal sealing has been engineered in integral bilayer scaffolds (42), while nanozyme-functionalized bilayers and mechanically graded hydrogels add zone-specific biological or mechanical cues (43, 44). For talar reconstruction, the lateral cartilage interface and the bone-facing interface should therefore be assessed separately under relevant loading conditions.

Architectural complexity does not guarantee clinical practicality. A multilayer scaffold may reproduce elements of native osteochondral organization, but this advantage is limited if the implant is difficult to trim, seat, or secure in a confined talar defect. A simpler construct may be more useful when it can be delivered reproducibly and provides dependable osseous support. Surgical handling and surface congruity are therefore as important as biomimetic architecture.

Continuous gradients and layered constructs offer different tradeoffs. A continuous gradient may reduce an abrupt transition across the osteochondral unit, whereas a layered implant may be easier to manufacture, handle, or combine with bone grafting. The relevant comparison is not which design appears more biomimetic, but whether it improves load transfer, edge stability, and osseous anchorage in a reproducible manner.

### 4.3 Bioactive modulation

Once delivery and mechanical stability are established, bioactive cues may be used to influence cell recruitment and the local repair environment. Exosome-loaded adhesive hydrogels and patterned drug or gene delivery systems illustrate this concept in cartilage and osteochondral models (51, 52).

Immunomodulation remains preclinical. In a rabbit osteochondral model, a 3D-printed PRP-GelMA construct was associated with improved repair and a shift toward an M2 macrophage phenotype (53). This finding does not establish talus-specific efficacy. Bioactive functions should be judged by whether they add measurable benefit without compromising delivery, retention, fatigue resistance, or osteochondral anchorage.

### 4.4 Clinical evidence for matrix- and scaffold-assisted talar repair

Human studies have established clinical feasibility for several matrix- and scaffold-assisted approaches, although the evidence does not support choosing treatment on material type alone. Early one-stage procedures using bone marrow-derived cells or hyaluronic acid carriers reported clinical improvement (54, 55). Longer follow-up of hyaluronic scaffold transplantation with bone marrow aspirate concentrate suggests that improvement can persist in selected patients (56). Generalization remains limited by nonrandomized designs, evolving techniques, and selected lesion populations.

Autologous matrix-induced chondrogenesis and collagen-assisted procedures broadened this experience across arthroscopic and open surgery. Arthroscopic AMIC with autologous bone graft has been used for deeper osteochondral defects (26). Early- to medium-term cohorts, shoulder-lesion studies, and MRI follow-up report clinical improvement while also showing that symptoms and structural repair do not always progress in parallel (27–29). Open reconstruction, revision, deep-lesion, and long-term cohorts extend the evidence to more demanding settings, but differences in lesion size, cystic depth, containment, concomitant procedures, rehabilitation, imaging, and follow-up complicate comparison (30–33).

Comparative studies are not uniformly favorable. In a prospective cohort of selected talar shoulder lesions, AMIC was associated with better AOFAS, VAS, and Tegner scores and lower reoperation and failure rates than microfracture, while MOCART scores did not differ significantly (28). A propensity-matched cohort found similar 1-year clinical score improvement after atelocollagen-induced chondrogenesis and microfracture, although satisfaction and several quality-of-life domains favored the augmented group (57). A multicenter randomized trial then found a higher 2-year MOCART score with atelocollagen augmentation but no statistically significant between-group differences in VAS, HSS, or AOFAS scores (58). Chitosan-blood augmentation did not improve overall clinical or MRI outcomes compared with microfracture alone (59). Matrix or scaffold addition therefore cannot be assumed to improve outcome independently of lesion characteristics and the accompanying surgical procedure.

Results with other platforms are similarly mixed. A multicenter randomized comparison of arthroscopic strategies and a four-patient pilot series using a biphasic aragonite implant illustrate technical and clinical feasibility but do not establish material-specific efficacy (60, 61). In deep lesions, a cohort comparing cancellous bone grafting with vs. without a collagen scaffold found no additional clinical benefit from scaffold use, and the MOCART score was slightly lower in the scaffold group (62). Accordingly, Table 1 displays lesion size/depth, lesion phenotype, subchondral treatment, instability/alignment, and comparator information where reported, so that clinical outcomes can be interpreted against the mechanical context of each cohort.

Recent studies further show why lesion morphology, subchondral treatment, and the treatment package matter. A 2026 retrospective case series of 36 patients treated with an arthroscopically applied autologous platelet-rich fibrin scaffold reported improvements in pain and functional scores, with more than 80% returning to sport, but the absence of a comparator prevents attribution of the improvement to the scaffold itself (135). Medial cystic lesions were associated with lower sports activity and inferior radiological outcomes than lateral lesions after marrow stimulation (113). Precision micro-drilling produced faster early recovery and better MOCART scores than conventional microfracture, indicating that the method of subchondral access can influence recovery and repair quality (114). Recent AMIC cohorts also report sustained improvement and return to sport in selected patients, while differences in lesion selection, imaging, follow-up, and subchondral management complicate comparison across series (106, 107, 109).

Direct comparisons of AMIC with microfracture have not shown uniform superiority of matrix augmentation (93). In more structurally demanding lesions, studies combining matrix techniques with spongiosa grafting or biomimetic scaffolds have reported favorable clinical and imaging outcomes (75, 81, 85). Additional series indicate that instability, shoulder location, age group, body weight, and lesion size may modify outcome (88, 97, 100, 105, 115). Studies of EFAS/MOCART correlations, complications, blood-concentrate augmentation, and all-arthroscopic AMIC further demonstrate why symptoms, imaging, safety, and return to activity should remain separate endpoints (108, 110, 111, 116–118).

Outcome definition and cohort composition remain major limitations when interpreting this literature. Functional scores can improve despite incomplete MRI repair, while short follow-up may miss later structural failure. Results from a contained lesion should not be generalized automatically to shoulder or cystic defects, and a mixed cohort can obscure benefit or failure within a particular lesion subtype. Clinical reports should therefore separate symptom improvement, structural integration, return to activity, reoperation, and long-term failure, while also reporting lesion phenotype and subchondral treatment.

### 4.5 Biofabrication and emerging spatial architectures

Most advanced spatial biofabrication platforms discussed below have not been evaluated specifically in talar lesions. Their value for this review is therefore not that they demonstrate ankle efficacy, but that they test distinct ways of organizing mechanical and biological functions across an osteochondral construct. The representative studies were considered by architectural function rather than by material novelty alone: continuous or zonal gradients, discrete biphasic or multilayer constructs, directional spatial organization, and fabrication or fixation strategies that determine whether a construct can be manufactured, positioned, and retained. This distinction is important because a design principle may be transferable to the talus even when the biological outcome was demonstrated in a femoral or generic osteochondral model.

Gradient and zonal systems address the abrupt transition between compliant cartilage and mechanically supportive subchondral bone. A 3D-printed osteochondral scaffold incorporating decellularized cartilage extracellular matrix in the cartilage zone promoted host-cell recruitment and cartilage-matrix formation in a rabbit defect model (119). Trilayered hydrogels have extended this concept by separating an upper cartilage-like layer, a tidemark-like intermediate layer, and a stiffer bone-facing layer with different mechanical and biological cues (120). More recently, bilayer hydrogels combining a soft cartilage-facing region with a nanohydroxyapatite-reinforced bone-facing region and directional microchannels have coupled a through-thickness mechanical gradient with spatial guidance (127). A 2026 3D-bioprinted bilayer GelMA/hydroxyapatite scaffold functionalized with miR-140-5p-modified mesenchymal stem cells combined zone-specific material properties with cell-based regulation and improved osteochondral repair in a rabbit femoral-condyle model (136). These studies support the principle that the cartilage-bone transition can be deliberately distributed rather than represented by a single homogeneous material. For a deep or cystic talar lesion, however, the relevant test is whether such a gradient improves vertical load transfer and resists bone-side subsidence without producing swelling-related surface step-off or interfacial failure under cyclic loading.

Discrete biphasic and multilayer constructs provide a related but more modular strategy. Earlier work used seamless biphasic hydrogels, hydrogel-mineral composites, bilayered PLGA scaffolds, additively manufactured multilayer scaffolds, PLGA/PLGA-HAp composites, and paired decellularized cartilage/decalcified bone matrices to create separate cartilage- and bone-oriented regions (128, 129, 131–134). Recent hierarchical biphasic scaffolds have added spatially restricted immunomodulatory and anabolic functions to this structural division (126). The advantage of a layered design is that the cartilage-facing and bone-facing phases can be optimized separately; the limitation is that every added interface can become a site of delamination, manufacturing variability, or geometric mismatch. This tradeoff is particularly relevant in the talus, where a construct must be trimmed and seated within a confined defect while maintaining a flush articular surface and dependable osseous support.

Spatial organization can also guide repair within a layer rather than only across cartilage and bone. Radially oriented collagen scaffolds with SDF-1 have been used to direct cell homing and tissue organization (130), while directional microchannels provide a means of controlling transport and cellular orientation within a mechanically graded hydrogel (127). Biofabrication with preformed cartilage microtissues offers another form of spatial control: rapidly degrading supporting hydrogels can temporarily localize microtissues into a defined three-dimensional geometry while allowing subsequent tissue fusion (123). These approaches are relevant to the talus because defect geometry is small and irregular, but their usefulness will depend on whether the intended architecture is preserved after implantation, swelling, early motion, and repetitive compression and shear. Spatial organization that improves histology but is lost at the defect edge or under load would have limited translational value.

Manufacturing and fixation are therefore part of biofabrication rather than downstream practical details. The 3D-printed template used in zonal osteochondral scaffolds provides geometric definition and mechanical support (119), whereas rapidly degrading support hydrogels illustrate how a temporary bioink can enable assembly without becoming the long-term load-bearing phase (123). Adhesive hydrogel studies on human osteochondral interfaces further show that fixation can be engineered directly into a material, but also that hydration materially influences interfacial performance (124). Wood-derived hydrogels, nanoenabled collagen-polylactide scaffolds, and acellular cartilage-matrix/thermosensitive hydrogel systems broaden the available material and biological functions (121, 122, 125), yet multifunctionality does not by itself solve arthroscopic delivery, rim retention, sterilization, reproducibility, or surface congruity. These parameters should be considered alongside biological readouts when judging whether an advanced construct is realistically translatable.

For talar repair, the contribution of biofabrication is best judged against a defined lesion problem. A small contained lesion may favor a conformal or adhesive *in situ* system; a shoulder or uncontained lesion places greater emphasis on edge fixation and resistance to shear; and a deep cystic lesion may justify a graded or biphasic architecture that provides bone-side support. Revision lesions add the need for reliable integration with a compromised bed, whereas persistent instability or malalignment remains a joint-level problem that should be corrected rather than delegated to the biomaterial. Future preclinical studies should therefore compare spatially organized constructs with simpler controls and identify what the added architecture actually contributes—for example, improved load transfer, edge stability, cell guidance, or osteochondral integration—while also reporting implantation accuracy, wet-field retention, cyclic deformation, interface failure, degradation under load, sterilization, and manufacturing reproducibility. This would make biofabrication a testable lesion-specific strategy rather than a descriptive increase in material complexity.

## 5 Discussion

The principal translational challenge is not a shortage of candidate biomaterials, but whether their properties address the failure modes that matter in the ankle. Clinical translation depends first on accurate placement, retention at the defect margin, and support across the osteochondral interface during repetitive loading. Hydrogel-based systems may fail through washout, debonding, or fatigue, whereas structured scaffolds may be limited by poor seating, edge mismatch, or inadequate integration.

Injectability, adhesion, and mineralization address different parts of this problem. Injectability is useful when it allows controlled filling of an irregular defect. Adhesion matters when it maintains contact with hydrated cartilage during fluid exposure and motion. Mineralized or graded regions become relevant when subchondral support and load transfer are required. Calling a construct multifunctional is less informative than showing which of these specific problems it solves and under what loading conditions.

Advanced biological functions need to be interpreted in the same clinical context. Cell-recruitment cues, gene or drug delivery, and immune modulation may add value to selected constructs, but they do not remove the need for reliable handling and mechanical stability. Preclinical studies would be more informative if biological readouts were reported alongside wet-field handling, washout resistance, cyclic compression and shear, edge integration, degradation under load, and the location and mode of failure.

Biofabrication may be most useful when spatial complexity solves a specific talar problem rather than reproducing osteochondral zonation for its own sake. A gradient, multilayer, or directionally organized construct should therefore be compared with a simpler control to show what the added architecture contributes—more stable load transfer, less edge motion, better bone-side support, or more directed cellular and tissue organization. Conversely, a technically sophisticated construct that cannot be delivered through the intended surgical approach, maintain a flush surface, or tolerate repeated loading offers limited translational advantage. The current non-talar evidence supports these design hypotheses but does not establish clinical superiority in osteochondral lesions of the talus (119–134).

The clinical literature has a different but related limitation. Heterogeneity in lesion size, containment, cystic depth, grafting, instability correction, alignment, surgical approach, rehabilitation, imaging, and follow-up makes direct comparison difficult. Consistent reporting of these variables would make it easier to determine whether a matrix or scaffold adds value beyond marrow stimulation, bone grafting, fixation, or correction of the mechanical environment.

Clinical translation will depend on matching defined material or biofabrication properties to specific lesion problems rather than adding functions without a clear indication. Biomaterials studies can provide detailed chemistry, histology, and regenerative signaling, whereas orthopedic translation requires evidence that the construct can be delivered, seated, retained, integrated, and loaded without introducing a new mechanical problem. Accordingly, preclinical and clinical evaluation should report manufacturing reproducibility, wet-field handling, interface failure, degradation under cyclic load, and clinically meaningful lesion-stratified outcomes alongside biological repair.

## 6 Conclusion

Hydrogel, scaffold, and biofabrication strategies for talar osteochondral repair should be evaluated against the anatomical and mechanical demands of the ankle rather than by material complexity alone. The most relevant requirements are controlled defect filling, stable wet-field retention, fatigue tolerance, and cartilage-bone anchorage, with the balance among them determined by lesion containment, subchondral depth, instability, and alignment.

Human evidence supports matrix- and scaffold-assisted repair in selected patients, but no material strategy has shown consistent superiority across talar lesion phenotypes. Further progress will require lesion-specific clinical reporting together with preclinical assessment of handling, edge stability, cyclic loading, bone-side integration, degradation under load, and failure mode. Bioactive functions are most convincing when they improve a construct whose basic mechanical performance has already been established.
